# In Vitro Analysis of the Mechanical Properties of Hypoallergenic Denture Base Resins

**DOI:** 10.3390/ma15103611

**Published:** 2022-05-18

**Authors:** Sebastian Hinz, Tobias Bensel, Wolfgang Bömicke, Arne F. Boeckler

**Affiliations:** 1 Department of Prosthodontics, Martin-Luther-University Halle-Wittenberg, Magdeburgerstraße 16, 06112 Halle, Germany; info@zahnarzt-am-rain.de (T.B.); arne.boeckler@web.de (A.F.B.); 2 Department of Prosthetic Dentistry, University Hospital Heidelberg, University of Heidelberg, Im Neuenheimer Feld 400, 69120 Heidelberg, Germany; wolfgang.boemicke@med.uni-heidelberg.de

**Keywords:** dental materials, mechanical tests, polymethyl methacrylate, in vitro study

## Abstract

The development of hypoallergenic denture resins is key to the treatment of patients with allergies to polymethyl methacrylate (PMMA). In this study, the in vitro mechanical properties of hypoallergenic and PMMA denture base resins were compared. Ninety-six test specimens of hypoallergenic denture base resins (Polyan Plus^®^, Sinomer, TMS Acetal Dental, Erkocryl) and 72 test specimens of PMMA-based denture base resins (Paladon 65, PalaXpress, SR-Ivocap) were fabricated. The flexural strength, elastic modulus, compressive strength, macro- and microhardness, average roughness, water absorption, and water solubility of the resins were measured. None of the hypoallergenic denture resins matched all the mechanical properties of the PMMA resins. Polyan Plus^®^ and TMS Acetal Dental were closest to matching the mechanical properties of the PMMA resins, and TMS Acetal Dental had some superior properties. Consequently, Polyan Plus^®^ and TMS Acetal Dental hypoallergenic resins are recommended for further investigation as potential alternatives to PMMA resins for the fabrication of removable dentures.

## 1. Introduction

Polymethyl methacrylate resins are (PMMA) almost always used as the base material for prostheses due to ease of processing, low cost, easy reparability, and their overall favorable physical and chemical properties [1]. PMMA resins have the property of absorbing and releasing water. This exposes the material to internal stresses, which can lead to instability, cracking and the eventual fracture of the prosthesis. Therefore, water absorption and solubility should be as low as possible. There is a correlation between residual monomer and water absorption. If residual monomer is present, less monomer conversion takes place, which can lead to increased water absorption and release [1,2,3].

PMMA denture base resins can trigger allergic contact dermatitis, such as type IV hypersensitivity or delayed reactions, in patients during prosthodontic procedures [4,5,6,7,8,9,10,11,12,13,14]. The polymerization of methyl methacrylate (MMA) includes the addition of stabilizers such as hydroquinone and initiators such as dibenzoyl peroxide (BPO). However, hardened denture base resins still contain traces of unpolymerized MMA and BPO, which cause type IV allergic reactions as they are released from PMMA during use [15,16,17,18]. Each resin is more or less cytotoxic. It depends, among other factors, on the amount of residual monomer [2]. Different types of resins have different cytotoxic effects on human cells. This has been demonstrated, for example, via the gene expression of p53, p21 and bcl2 [1]. These allergens affect both patients and dental personnel (dentists, dental assistance personnel, and dental technicians) [19,20,21,22,23,24]. To make prosthodontic treatment safer for patients with proven incompatibility to these materials, researchers have invented commercially viable hypoallergenic denture base resins. Their formulations either do not contain MMA or contain a negligible amount of it [3,25,26]. In hypoallergenic resins, MMA is replaced, for example, by diurethane dimethacrylate, polyurethane, polyethylene terephthalate, polyethylene terephthalate and polybutylene terephthalate. This has an influence on the physical and chemical properties of the hypoallergenic denture base resins [25]. Furthermore, the processing method of the different denture base materials has an influence on their material properties [27].

To function as an equivalent alternative to PMMA, hypoallergenic denture base resins must possess comparable mechanical properties. In this study, the relevant material–mechanical properties of hypoallergenic denture base resins were compared with those of established PMMA resins to expand on discussions in the literature and fill data gaps. Safe treatment options for dentists that involve the use of commercial hypoallergenic denture resins are currently still limited.

Therefore, we performed an in vitro study to determine the flexural strength, elastic modulus, compressive strength, macro- and microhardness, surface roughness, water absorption, and water solubility of four hypoallergenic denture base resins and compared them with those of three conventional PMMA denture base resins. We hypothesized that the hypoallergenic denture base resins would have comparable mechanical properties to the PMMA denture base resins.

## 2. Materials and Methods

### 2.1. Test Specimens

We fabricated 168 specimens from seven different denture base reins (n = 24 per denture base resin group) (Table 1). the following four hypoallergenic denture base resins were used in the experimental group: TMS Acetal Dental (Pressing Dental S.R.L, Dogana, San Marino), Erkocryl (Erkodent Erich Kopp GmbH, Pfalzgrafenweiler, Germany), Polyan Plus^®^ (Polyapress GmbH, Altkirchen, Germany), and Sinomer (ALLDENT AG, Rugell, Liechtenstein). The following three PMMA-based denture resins were used in the control group: Paladon 65 (Heraeus Kulzer GmbH, Hanau, Germany), PalaXpress (Heraeus Kulzer GmbH, Hanau, Germany), and SR-Ivocap (Ivoclar Vivadent GmbH, Ellwangen, Germany). All investigated denture base resins had ensured market availability in Europe. According to DIN EU ISO Norm 3167:2003, the test specimens were prismatically designed with dimensions of 64 × 40 × 4 mm. TMS Acetal Dental, Erkocryl, and Polyan Plus^®^ specimens were fabricated by injection molding; the Sinomer, PalaXpress, and SR-Ivocap specimens were produced by a high-pressure injection process; and Paladon 65 was fabricated by casting. Test specimen surfaces were ground and polished using SiC paper with grit sizes of 220, 320, 800, 1200, and 2400 (RotoPol-35, Struers GmbH, Willich, Germany). All test specimens were stored at room temperature (23 °C) and 50% relative humidity (WTC Binder, Tuttlingen, Germany) until the experiment.

### 2.2. Elastic Modulus and Flexural Strength

The elastic modulus and flexural strength of n = 6 test specimens per denture base resin group were measured by three-point bending tests according to DIN EN ISO 178:2003 at room temperature (23 °C) and 50% relative humidity. The tests were performed on a universal testing machine (UTM; ZWICKI TMZW, Zwick GmbH & Co. KG, Ulm, Germany). The specimens were mounted on two 5 mm diameter support posts, 50 mm apart. The abutments and plunger had a diameter of 5 mm each. The elastic modulus and flexural strength were determined at a crosshead speed of 5 mm/min and calculated automatically using testing and calibration software (testXpert 7.0, Zwick GmbH & Co. KG, Ulm, Germany).

### 2.3. Compressive Strength

The compressive strength was determined for n = 8 test specimens of each denture base resin according to DIN EN ISO 604:2003-02. The specimens were cut into cuboids with dimensions of 10 × 10 × 4 mm and loaded on the UTM until fracture at a constant test speed of 1 mm/min. The maximum force at fracture was recorded using testXpert 8.1 software.

### 2.4. Macrohardness

The macrohardness of n = 2 test specimens from each denture base resin group was measured by testing the indentation hardness according to DIN EN ISO 2039-1 using the Instron Wolpert-Macro Hardness K-Testors 2524 (Wolpert Wilson Instruments, Pfungstadt, Germany). Test specimens with dimensions of 64 × 40 × 4 mm were loaded with steel spheres exerting forces of 132 N, 358 N, and a maximum test force (diameter = 5 mm). Ten hardness measurements were taken for each specimen, with 10 mm between each indentation and the specimen margin. To ensure the validity of the hardness values, the test load was selected such that the corresponding penetration depth of the indenter into the test specimen after 30 s was within the range of 0.15–0.35 mm.

### 2.5. Microhardness

The microhardness of n = 1 test specimens with dimensions of 64 × 40 × 4 mm for each of the investigated denture base resins was measured using a microhardness test device (Fischerscope H 100C XYp, Helmut Fischer GmbH, Sindelfingen, Germany) with a Vickers diamond pyramid indenter (opening angle = 136°). The tests were performed according to DIN EN ISO 14577-1–3. The test area was selected microscopically (Video-Measuring and Inspection system VMZM-40, 4H-Jena engineering, Jena, Germany). Every test specimen was loaded at a rate of 40 mN/s up to a maximum load of 1000 mN. The microhardness was measured at ten independent areas on each test specimen. The depth of the impression and stress of the indenter were registered simultaneously and displayed graphically using testing software (WIN-HCU-Software, Fischer, Sindelfingen, Germany).

### 2.6. Average Roughness

For the average roughness investigation, n = 2 test specimens were used per denture base resin group. The average roughness was measured using a surface-measuring device (Perthometer PGK, Mahr GmbH, Göttingen, Germany) and an evaluation device (Perthometer S3P, Mahr GmbH, Göttingen, Germany) according to DIN EN ISO 4287, 4288, and 4760. The length of the test track was 5.6 mm. The total measuring length was 4.0 mm. The calibrated total measuring deviation of the tactile incision technique was set to <1.5%. Five parallel measurements were performed on each test specimen with a cut-off wavelength of 0.8 mm.

### 2.7. Water Absorption and Water Solubility

Water absorption and solubility were investigated for n = 5 test specimens per denture base resin group according to DIN EN ISO 1567. All test specimens were stored in a desiccator and aerobically incubated at 37 °C (WTC Binder, Tuttlingen, Germany) for 24 h. Subsequently, the constant mass was weighed, and the volume of the test specimen was measured. A deviation of ≤0.2 mg was considered a constant mass. The drying process was repeated until this was achieved. The number of passes required to achieve this was not recorded. The specimens were then immersed in distilled water and aerobically incubated at 37 °C (WTC Binder, Tuttlingen, Germany) for seven days. This process was repeated until a mass difference was detected.

### 2.8. Statistical Analysis

The means and standard deviations of the mechanical properties were calculated, and the results are presented as bar plots. The data were analyzed using a one-way analysis of variance and the Bonferroni post hoc test (SPSS 25.0 for Windows (SPSS Inc., Chicago, IL, USA)). The level of significance was set to 5% (*p* ≤ 0.05).

## 3. Results

### 3.1. Flexural Strength

The flexural strengths ranged from 71.4 ± 8.5 MPa (Sinomer—minimum) to 136.1 ± 10.7 MPa (Polyan Plus^®^—maximum). Only Sinomer exhibited values below the comparative level of the PMMA denture base resins (92.8–120.2 MPa). Of the remaining hypoallergenic denture base resins, Polyan Plus^®^ (136.1 ± 10.7 MPa) and TMS Acetal Dental (123.9 ± 13.9 MPa) exceeded the comparative level in terms of flexural strength (Table 2). As per the post hoc test, Polyan Plus^®^ (*p* = 0.001) and Sinomer (*p* < 0.001) had significantly different flexural strengths compared with the control group (Table 3).

### 3.2. Elastic Modulus

The elastic moduli ranged from 2208.0 ± 72.0 MPa (Sinomer—minimum value) to 3311.0 ± 248.0 MPa (TMS Acetal Dental—maximum value). Only Sinomer exhibited an elastic modulus that was below the comparative level of the PMMA denture base resins. TMS Acetal Dental (3311.0 ± 248.0 MPa) exceeded the comparative level. The values of the remaining denture base resins were comparable (Table 2).

As per the post hoc test, Polyan Plus^®^ (*p* = 0.001) and Sinomer (*p* < 0.001) displayed notably different flexural strengths compared with the control group. Sinomer (*p* < 0.001), TMS Acetal Dental (*p* = 0.046), and Erkocryl (*p* = 0.040) had significantly different elastic moduli than the control groups (Table 3).

### 3.3. Compressive Strength

The compressive strength values ranged from 99.8 ± 8.7 MPa (Erkocryl—minimum) to 216.9 ± 33.5 MPa (Sinomer—maximum). The comparative level of the PMMA denture base resins was 103.8–150.1 MPa. The strength of Sinomer (216.9 ± 33.5 MPa) was above the comparative level of the PMMA resins. Only Erkocryl (99.8 ± 8.7 MPa) exhibited strength values of below the level of the PMMA denture base resins (Table 2). Sinomer (*p* < 0.001) and Erkocryl (*p* = 0.037) had vastly different strengths compared with the control group (Table 3).

### 3.4. Macrohardness

The macrohardness values of the test specimens ranged from 64.0 ± 26.0 N/mm^2^ (Sinomer—minimum) to 93 ± 19.1 N/mm^2^ (TMS Acetal Dental—maximum). The macrohardness values of the hypoallergenic resins were all below the comparative level of the PMMA resins (Table 2). The values for Polyan Plus^®^ (*p* < 0.001), Sinomer (*p* < 0.001), TMS Acetal Dental (*p* < 0.001), and Erkocryl (*p* < 0.001) demonstrated significant differences with the control group (Table 3).

### 3.5. Microhardness

The microhardness values of the test specimens ranged from 129.6 ± 8.5 N/mm² (Sinomer—minimum) to 200.9 ± 8.7 N/mm² (Polyan Plus^®^—maximum). The values of all tested hypoallergenic resins, except for Sinomer and Erkocryl, were within the comparative range of the PMMA resins (Table 2). The microhardness results of Polyan Plus^®^ (*p* < 0.001), Sinomer (*p* < 0.001), and Erkocryl (*p* < 0.001) (Table 3) were significantly different from those of the control group.

### 3.6. Average Roughness

The average roughness values of the test specimens ranged from 0.02 ± 0.01 µm (Erkocryl—minimum) to 0.38 ± 0.02 µm (TMS Acetal Dental—maximum). TMS Acetal Dental (0.38 ± 0.02 µm) and Sinomer (0.16 ± 0.04 µm) showed data above the comparative level of the PMMA resins while the Erkocryl (0.02 ± 0.01 µm) and Polyan Plus^®^ (0.03 ± 0.01 µm) values were below the comparative level (Table 2). Sinomer (*p* < 0.001) and TMS Acetal Dental (*p* < 0.001) showed significant differences compared to the control group (Table 3).

### 3.7. Water Absorption

The water absorption values of the test specimens ranged from 19.02 ± 2.06 μg/mm^3^ (PalaXpress—minimum) to 25.90 ± 2.63 μg/mm^3^ (Erkocryl—maximum). The water absorption values of Sinomer, TMS Acetal Dental and Polyan Plus^®^ were at or above the comparative level of the PMMA resins (Table 2).

### 3.8. Water Solubility

The water solubility values of the test specimens ranged from 0.99 ± 0.10 μg/mm^3^ (Sinomer—minimum) to 1.45 ± 0.22 μg/mm^3^ (TMS Acetal Dental—maximum). With the exception of Sinomer, all the tested hypoallergenic denture base resins were comparable with the PMMA resins (Table 2). The post hoc test demonstrated that neither the water absorption nor the water solubility results of the investigated hypoallergenic denture base resins showed significant differences with the control group (Table 3).

Polyan Plus^®^ and TMS Acetal Dental exhibited the best mechanical properties among the investigated hypoallergenic resins. Moreover, their mechanical properties were comparable to those of the PMMA-based denture base resins. Polyan Plus^®^ exhibited macrohardness data of below the comparative level of the PMMA resins. The highest microhardness values were detected for Polyan Plus^®^. The remaining parameters of Polylan Plus^®^ were within (water solubility/absorption) or below the comparative level (average roughness). TMS Acetal Dental exhibited increased values of average roughness measurements and macrohardness values below the comparative level. Erkocryl fulfilled the requirements in four of the investigated material–mechanical parameters. Sinomer satisfied only two required material–mechanical properties (Table 4). The one-way ANOVA revealed the significant flexural strength (*p* < 0.001), elastic modulus (*p* < 0.001), compressive strength (*p* < 0.001), macro- and microhardness (*p* < 0.001), average roughness (*p* < 0.001), water absorption (*p* = 0.014), and solubility (*p* < 0.001) (Table 3) of the denture base resin.

## 4. Discussion

This in vitro study classified the material–mechanical properties of hypoallergenic denture base resins and compared them with PMMA-based denture base resins. The latter are used in prosthodontics. PMMA denture base resins have been used in several in vitro studies as reference materials for evaluating the material–mechanical properties of denture base resins [3,25,26]. For each test, the range of values to be achieved by the hypoallergenic resin materials was set to the minimum and maximum values obtained for their PMMA counterparts. Given the general lack of in vitro data on the mechanical properties of hypoallergenic denture resins, some of the results could not be compared with those in the literature.

If the range of mechanical properties of PMMA materials was taken as the benchmark, Sinomer would not meet this standard for either flexural strength (71.4 MPa, Table 2) or elastic modulus (2208 MPa, Table 2). The obtained values are both confirmed and contradicted by other studies (Pfeiffer et al. [26] (flexural strength 72.3 MPa, elastic modulus 1720 MPa) vs. Lassila and Vallitu [28] (flexural strength 85.8 MPa, elastic modulus 2730 MPa). The flexural strength of the denture base resin was reduced, even if the water absorption increased. This could indicate the possible effect of the addition of plasticizer and must be explored further [29,30].

The highest flexural strength values were determined for Polyan Plus^®^ (136.1 MPa), which was confirmed by other studies (Table 2) [26]. TMS Acetal Dental (123.9 MPa) also showed an increased flexural strength (Table 2). Therefore, we assumed that the fabrication process could have led to high flexural strength, as the mechanical properties of denture base resins heavily rely on the processing method [31,32]. Both the Polyan Plus^®^ and TMS Acetal Dental hypoallergenic denture base resins were manufactured using injection molding.

Erkocryl did not reach the necessary compressive strength (99.8 MPa) (Table 2). The compressive strength values of the remaining hypoallergenic denture base resins were comparable to those of the PMMA counterparts (Table 2). Sinomer exhibited substantially higher compressive strength (216.0 MPa) values (Table 2). The values for TMS Acetal Dental (116.6 MPa) were at the lower bound of the comparative level (Table 2). No recent data regarding these results are available in the literature for comparison.

All the hypoallergenic denture base resins had microhardness values of below the comparative level. Sinomer (64 N/mm²) showed the smallest microhardness (Table 2). Therefore, we concluded that Sinomer is heterogeneous.

The microhardness values of Sinomer (129.6 N/mm²) and Erkocryl (142.9 N/mm²) were below the comparative level (Table 2). This may lead to an early material breach. Except for Polyan Plus^®^ (200.9 N/mm²), which had a mean microhardness value above the comparative level, the hypoallergenic resins had scattered microhardness values in the comparative band of the PMMA counterparts (Table 2).

Polyan Plus^®^ (0.03 µm) and Erkocryl (0.02 µm) had similar or lower average roughness values compared to the control group of PMMA denture base resins (Table 2). The remaining hypoallergenic resins exhibited substantially higher average roughness values. TMS Acetal Dental (0.38 µm) demonstrated the lowest surface quality of all the investigated denture base resins (Table 2).

Polyan Plus^®^ (19.36 μg/mm^3^) exhibited less water absorption than its PMMA counterpart (Table 2).

Sinomer (0.99 μg/mm^3^) and Polyan (1.03 μg/mm^3^) had the smallest water solubilities (Table 2). Sinomer had a lower solubility than the PMMA resins. However, it showed higher solubility in comparative studies [28]. The water solubility of TMS Acetal Dental (1.45 μg/mm^3^) was at the upper limit of the comparative level. The water solubilities of Polyan Plus^®^ and Erkocryl were within the comparative level of the PMMA resins (Table 2). Higher water solubilities could be caused by a reduced length and degree of polymerization [28]. Nevertheless, the water solubility values in this study were significantly scattered. This can be reasonably explained by the differences in measurements between the weight of the denture base resins at the drying and re-drying periods owing to measurement inaccuracy.

Water absorption could affect the mechanical properties of denture base resins because water has an effect similar to that of the residual monomer as a plasticizer [33,34,35]. In turn, the presence of residual monomers in denture base resins could lead to increased water absorption and water solubility [36]. However, according to the manufacturer guidelines of the investigated hypoallergenic denture base resins, no significant residual monomer components were included. In this study, Sinomer (25.01 μg/mm^3^) and Erkocryl (25.9 μg/mm^3^) had greater water absorption values than the PMMA denture base resins (Table 2).

Thus far, no definitive causes have been identified for this trend. Therefore, the influences of processing, such as forming, mold filling, and hardening must be explored. Relations between the material properties (flexural strength, elastic modulus, and water absorption) could be empirically determined for Polyan Plus^®^ and TMS Acetal Dental. These hypoallergenic resins exhibited comparatively low water absorption and high flexural strength and elastic moduli. This relationship was not observed for Sinomer.

The limitations of this study are that the hypoallergenic denture base resins were compared with the range of values for PMMA denture base resins. A convenience sample was chosen and the sample size was based on previous studies [3,25,26]. That represents a weakness of this study. However, we did not confirm the quality of the PMMA specimens used to establish the comparative range.

## 5. Conclusions

Mechanical tests revealed that the hypoallergenic denture resins did not meet the standards of the PMMA resins, in that none of the resins met all the requirements of the material tests to a comparable degree. Polyan Plus^®^ and TMS Acetal Dental exhibited an acceptable flexural strength, elastic modulus, and compressive strength when compared to PMMA denture base resins as the benchmark. These two hypoallergenic resins can be recommended for use as denture base materials. Values of Erkocryl were lower than the values of the PMMA denture base resins for compressive strength, macrohardness, microhardness and water absorption. Sinomer showed even worse physical properties overall. This was compounded by the poorer average roughness. This in turn could be a disadvantage for the hygienic properties of the denture base. With a few limitations, this study shows that some market-available hypoallergenic denture resins could be recommended for some of their mechanical properties, but that further improvements are necessary to ensure hypoallergenic denture resins meet the standard of PMMA denture base resins. Further in vivo studies should verify the clinical use of improved hypoallergenic denture base resins.

## Figures and Tables

**Table 1:** Overview of the consistencies of the investigated denture base resins.

Trade Names	Main Components	Manufacturer	Processing Method	Polymerisation Process
**TMS Acetal Dental**	polyoxymethylene	Pressing Dental S.R.L, Dogana, San Marino	injection moulding	- Pressing system - Melting temperature: 220 °C - Melting time: 20 min and 4 bar pressure - Heating time: 2–5 min after the injection - Cooling time: 20–40 min
**Erkocryl**	polymethyl methacrylate containing small amounts of butyl acrylate	Erkodent Erich Kopp GmbH, Pfalzgrafenweiler, Germany	injection moulding	- injection moulding machine at a temperature from 250–290 °C - in a cold mould - plates of 120 mm diameter
**Polyan Plus** ^**®**^	modified methyl methacrylate	Polyapress GmbH, Altkirchen, Germany	injection moulding	- preheating at 260 °C for 15–17 min - injection under the pressure of 9.5 bar within 0.25 s into the hollow form
**Sinomer**	Acrylic polymer of methyl methacrylate and polyfunctional oligomers based on acrylates and urethane	ALLDENT AG, Rugell, Liechtenstein	injection	- in a water bath: 40 min at 100 °C and 10 min with a pressure of 3 bar - cool down at room temperature
**Paladon 65**	polymethyl methacrylate (PMMA)	Heraeus Kulzer GmbH, Hanau, Germany	stuff press procedure	- in a water bath: heating to 70 °C for 30 min - stabilising the temperature for 30 min - increasing the temperature of 100 °C within 20 min - stabilising the temperature for 30 min - slow cooling down
**PalaXpress**	methacrylate-copolymer (PMMA)	Heraeus Kulzer GmbH, Hanau, Germany	injection	- in a water bath: 15–30 min at 55 °C with a pressure of 2 bar
**SR-Ivocap**	polymethyl methacrylate (PMMA)	Ivoclar Vivadent GmbH, Ellwangen, Germany	Injection	- in a water bath: 35 min at 100 °C and 6 bar injection pressure - 30 min cool down (20 min under injection pressure)

**Table 2:** Means and standard deviations (in parentheses) of the investigated properties of denture base resins.

Trade Name	Flexural Strength [MPa]	Elastic Modulus [MPa]	Compressive Strength [MPa]	Macro-Hardness [N/mm²]	Micro-Hardness [N/mm²]	Average Roughness [μm]	Water Absorption [μg/mm^3^]	Water Solubility [μg/mm^3^]
**Hypoallergenic denture base resins**								
Polyan Plus^®^	136.1 (10.7)	3020 (142)	139.6 (5.3)	92.8 (20.7)	200.9 (8.7)	0.03 (0.005)	19.36 (2.62)	1.03 (0.16)
Sinomer	71.4 (8.5)	2208 (72)	216.9 (33.5)	64 (26)	129.6 (8.5)	0.16 (0.040)	25.01 (2.87)	0.99 (0.14)
TMS Acetal Dental	123.9 (13.9)	3311 (248)	116.6 (24.2)	93 (19.1)	171.5 (3.4)	0.38 (0.020)	22.00 (2.95)	1.45 (0.22)
Erkocryl	100.5 (16.2)	2522 (94)	99.8 (8.7)	91 (7.2)	142.9 (0.5)	0.02 (0.005)	25.90 (2.63)	1.41 (0.09)
**PMMA denture base resins**								
Paladon 65	120.2 (5.6)	3180 (43)	150.1 (44.9)	163.7 (4.2)	185.9 (1.7)	0.03 (0.004)	21.36 (3.89)	1.46 (0.13)
SR-Ivocap	92.8 (7.4)	2431 (133)	103.8 (13.5)	123.3 (2.9)	145.5 (3.8)	0.08 (0.060)	24.88 (1.33)	1.33 (0.06)
PalaXpress	113.9 (9.6)	3149 (52)	149.2 (35.9)	156.9 (3.4)	170.5 (1.2)	0.03 (0.008)	19.02 (2.06)	1.00 (0.14)

Comparative level of the PMMA denture base resins.

**Table 3:** Analysis of variance for denture base materials.

	df	Sum of Squares	Mean Squared	F	*p*-Value
Flexural strength					
Between groups	4	14,726.415	3681.604	20.108	<0.001
Within groups	37	6774.475	183.094		
Elastic modulus					
Between groups	4	4,650,718.365	1,162,679.591	15.324	<0.001
Within groups	37	2,807,354.611	75,874.449		
Compressive strength					
Between groups	4	73,561.177	18,390.294	20.899	<0.001
Within groups	58	51,036.527	879.940		
Macrohardness					
Between groups	4	73,650.485	18,412.621	51.229	<0.001
Within groups	65	23,362.314	359.420		
Microhardness					
Between groups	4	31,120.947	7780.237	48.332	<0.001
Within groups	65	10,463.313	160.974		
Average roughness					
Between groups	4	0.619	0.155	129.736	<0.001
Within groups	37	0.044	0.001		
Water absorption					
Between groups	4	148.221	37.055	3.743	0.014
Within groups	30	297.004	9.900		
Water solubility					
Between groups	4	0.905	0.226	6.053	<0.001
Within groups	30	1.122	0.037		

**Table 4:** Fulfilment of mechanical properties of investigated hypoallergenic denture base resins (+ = Values above the comparative level of the PMMA denture base resins/ − = Values below the comparative level of the PMMA denture base resins).

Trade Name	Polyan Plus^®^	Sinomer	TMS Acetal Dental	Erkocryl
Flexural strength	+	−	+	+
Elastic modulus	+	−	+	+
Compressive strength	+	+	+	−
Macrohardness	−	−	−	−
Microhardness	+	−	+	−
Average roughness	+	−	−	+
Water absorption	+	−	+	−
Water solubility	+	+	+	+
Summary	7/8	2/8	6/8	4/8

## Data Availability

The data used to support the findings of this study may be acquired via an application to the Department of Prosthodontics, Martin-Luther-University Halle-Wittenberg, through Dr. Arne Boeckler, Department of Prosthodontics, University Hospital Halle, Magdeburger Straße 16, 06112 Halle, Germany.
